# The Feasibility and Challenges of Conducting Online Research to Examine Movement Behavior in Parents and Children During the COVID-19 Pandemic

**DOI:** 10.3389/fpubh.2021.720083

**Published:** 2022-01-07

**Authors:** Katherine Q. Scott-Andrews, Alison L. Miller, Thomas J. Templin, Rebecca E. Hasson, Leah E. Robinson

**Affiliations:** ^1^Child Movement Activity and Developmental Health Laboratory, School of Kinesiology, University of Michigan, Ann Arbor, MI, United States; ^2^Department of Health Behavior and Health Education, School of Public Health, University of Michigan, Ann Arbor, MI, United States; ^3^School of Kinesiology, University of Michigan, Ann Arbor, MI, United States; ^4^Childhood Disparities Research Laboratory, School of Kinesiology, University of Michigan, Ann Arbor, MI, United States

**Keywords:** physical activity, motor competence, perceived competence, COVID-19, online research

## Abstract

The global pandemic of COVID-19 shifted the methodology of this research project. The purpose of this perspective article is to discuss the feasibility and challenges of converting an in-person mixed methods study that examined associations among and beliefs about physical activity, motor competence, and perceived competence to an online format with parents and children during the COVID-19 pandemic. Recruitment was conducted through a University research registry, social media, and public listservs. All correspondence with participants was through email and secure platforms. Physical activity was assessed with accelerometers mailed to participants. Motor competence was assessed through participant-filmed trials of motor skills. Perceived competence was assessed with the Self-Perception Profile for Adults and Children delivered on Qualtrics. Semi- structured interviews to examine beliefs were conducted over Zoom. Approximately 200 families expressed interest in the study, 76 parent-child dyads consented and assented, and 61 parent-child dyads completed at least one component of the study. It is feasible to conduct online research that contributes to scientific knowledge and has potential advantages. However, various challenges need to be considered regarding the application of online research. These challenges included recruitment, the data collection process, and data quality. Future research needs to address these challenges by utilizing wide-reaching and diverse recruitment methods, easing participants' burden with technology, and developing motor competence and perceived competence assessments that can be administered online. The way research was conducted changed due to COVID-19 and adapting to and/or integrating online methods is both necessary and feasible, but modifications must be taken into consideration.

## Introduction

Physical activity is an effective way to promote overall health. It is well-established that there is a positive and favorable association between physical activity and numerous health indicators in children and adults (1, 2). Despite these direct health benefits, both children's and adult's physical activity levels are extremely low in the United States (3, 4). A potential contributing factor to low physical activity is low motor competence and perceived competence (5, 6). Motor competence is one's proficiency in a wide range of gross motor skills, including fundamental motor skills (FMS; i.e., locomotor and ball skills) (5). FMS are basic learned movement patterns or “building blocks” that do not occur naturally and are foundational to more complex movements, sports, and physical activities (7). Motor competence has been associated with physical activity in children (8–10) and adolescents (11–14). However, in adults, there is limited research examining motor competence, including FMS. Thus, the association with physical activity is not fully understood (15–18). Perceived competence refers to one's self-perception of their abilities (5, 6, 19). In children, perceived competence is significantly associated with motor competence (20–22) and physical activity (20, 21, 23), and has been found to mediate the association between motor competence and physical activity (11, 24, 25). In adults, perceived competence is significantly associated with actual motor competence (16, 17) and physical activity (17). Based on the literature, it is probable that low motor competence and perceived competence in children could be contributing to low levels of physical activity in adults. Still, to date, the research in this area is scarce.

Parents play an integral role in influencing health behaviors in their children, as they have the most contact hours in and control of their child's lives (26, 27). Research has shown that parents' physical activity is a significant predictor of their children's physical activity (26–30). However, to the authors' knowledge, no published research has investigated the association of motor competence between parent-child dyads. Lastly, few qualitative studies examine beliefs about physical activity (31–35), and none examining beliefs about motor competence. Exploring the associations of and beliefs about physical activity, motor competence, and perceived competence in parents and children was warranted.

The global pandemic of Coronavirus disease 2019 (COVID-19) and Executive Orders instituted across the United States (US) dramatically altered how we live, learn, and conduct research. The need to pivot from in-person to online research was essential and instantaneous (36, 37). Research using converted methodologies demonstrated feasibility, highlighted challenges, and provided recommendations (36). It is critical to examine methodologies across different fields to ensure high-quality research was conducted during COVID-19 and provide implications for future research (37). However, online research in motor development is unexplored. To the authors' knowledge, no studies had attempted to use online methodologies to assess motor skills nor perceived competence with parents and children before COVID-19. Conducting motor development research online faces unique challenges due to assessment feasibility and administration concerns. We used rapid-cycle evaluation (RCE) to convert an in-person mixed methods study to online.

The RCE approach entails identifying acute research problems and addressing them using contextually-appropriate methods (38). The phases of RCE are *preparation, problem exploration, knowledge exploration, solution development, solution testing*, and *implementation and dissemination* (38). For the *preparation* stage, the authors, experts in Kinesiology and Public Health, sought to identify the best way to alter this study while holding true to the original research questions and methods. The purpose of this perspective article was two-fold. First, we discussed the methods of adaptation (*problem exploration-solution development)* from in-person to online. Second, we discussed the feasibility/results *(solution testing)* and challenges/discussion of recruitment, data collection, and data quality of the converted online study that examined physical activity, motor competence, and perceived motor competence in parents and children.

It is important to examine and evaluate online research methodologies in motor development because we believe that as we emerge from COVID-19 motor development research will continue to be conducted online due to accessibility, potential research restrictions, and/or participants' apprehension of in-person research. The unprecedented times of COVID-19 provided a unique opportunity for online research to help advance the science and application of motor development research that will further advance the field.

## Methods

Following the RCE stages, the investigators adapted the original study using *problem exploration* and *knowledge exploration* (38). We had to identify the key problems and best solutions to convert an in-person study. In terms of recruitment, originally it was limited to local elementary schools. To adapt, the research team elected to recruit participants online through online social media (i.e., Facebook, Twitter, and Instagram), public listservs, and the University Research Registry for parent-child dyads. Inclusion criteria for children were children of all genders aged 8–11 years old, developmental ability to complete physical tasks, speak and understand English. Inclusion criteria for parents/legal guardians were: child's primary caregiver, ability to complete physical tasks, complete questionnaires, and speak and understand English. Participants had to be residents of Michigan and have access to filming devices and the internet. The decision to only include Michigan residents was due to individuals having more similar COVID-19 experiences since each state across the US had varying regulations and COVID-19 guidelines. Data collection took place from July-October 2020. All correspondence with participants took place through email and transfer of files (i.e., consents, assents, and data forms) was done through Box (Redwood City, CA) and Dropbox (San Francisco, CA), secure and confidential platforms. The original and adapted study was approved by the University of Michigan's Institutional Review Board (HUM00173043). The methods we adapted for each research variable are outlined below. See Table 1 for comparison of the original vs. adapted methods by research variable.

**Table 1:** Previous and adapted methods by research variable.

**Variables**	**Assessment**	**Original methods**	**Adapted methods**	**Data**
Demographics	Parent questionnaire	Parent questionnaire; pen and paper	Qualtrics	Age, gender, race, relation to child, socioeconomic status
Anthropometrics	Stadiometer and body composition analyzer	Researcher assessed	Qualtrics; self-reported	Height (cm), weight (lbs.), & BMI calculated of parent and child
Physical activity	Actigraph gt3x or gt3x+; 7-day wear protocol	Handed directly to participant	Mailed through USPS	Minutes/ day spent in MVPA & light, moderate, & vigorous activity
Motor competence	Process measures	Researcher administered full TGMD-3	Catch, jump, throw, and kick	Raw score (range 0–30)
	Product measures	Kick and throw speed, jump distance, and catch percentage	Jump distance and catch percentage	Cm and percentage
	Process and product	Researcher administered	Participant recorded videos	Z-scores
Perceived competence	Self-perception profile for adults and children; pictorial scale of perceived movement skill competence	Researcher administered; both assessments	Qualtrics; only Self-Perception Profile for Adults & Children	Perceived competence score; athletic competence, physical appearance, and global self-worth (range 1–4)
Beliefs	Semi-structured interview	Interview conducted in participant home	Zoom application	Themes

*MVPA, moderate to vigorous physical activity*.

### Demographics and Anthropometrics

Parents self-reported demographics and anthropometrics for themselves and their child through Qualtrics (Provo, UT).

### Physical Activity

Parent-child dyads were mailed Actigraph gt3x or gt3x+ trial-axis accelerometers (Actigraph LLC., Pensacola, FL), directions on how to wear the accelerometer for a 7-day wear period, a physical activity log to manually record wear time, and a prepaid return envelope. Mailings were distributed through the United States Postal Service (USPS).

### Motor Competence

The authors' created an online motor competence assessment. This assessment was adapted from existing and validated motor assessments; the Test of Gross Motor Development-3 (ICC = 0.97) (39) and product assessments that are sensitive discriminators (40–42). The first author (KQS) and two experts in Motor Development with over 10 years of experience in researching and administering motor skills developed and coded the assessment. Due to limitations with at-home administration, the online motor skills assessment only included four motor skills (i.e., catch, jump, kick, and throw). The catch, jump, kick and throw were assessed using the performance criterion of the Test of Gross Motor Development-3 (39) performance criterion. The product of catch percentage and jump distance were assessed through video software Dartfish (Pro6, Fribourg, Switzerland) (40–42). Aggregate process and product scores were created by standardizing the process and product measures and summing the created z-scores to develop the motor competence variable (41, 43, 44).

Parent-child dyads were emailed directions on how to perform the four different motor skills at home and film their performance. Parent-child dyads were instructed to gather the following equipment: a smartphone, tablet, or another filming device, a small ball/object to throw and catch, a larger ball or equivalent to kick, and optional measuring tape for the jump. Each motor skill had a corresponding multimedia demonstration available on YouTube. Multimedia demonstrations are an appropriate medium to use with the administration of motor assessments to ensure consistency in the demonstration (45).

The parent-child dyads were first instructed to watch the motor skills corresponding multimedia demonstration for each motor skill. Next, the directions instructed the parent-child dyads to perform one practice trial and watch the multimedia demonstration again. Then, the parent-child dyads performed two test trials for the throw and kick or five test trials for the catch and jump. This sequence was completed for each skill. This sequence was developed based on the standard protocol for administering the TGMD-3 (39) and has been used in administering product and process motor skills (41). In these standard protocols, participants perform one practice and two or five test trials for each motor skill. A skill demonstration is administered before the test trial, and if needed, again before the first test trial. The participant (i.e., the parent or child) not performing the motor skill was instructed to film the other participant's performance. Once all four motor skills were performed and filmed by both the parent and child, they uploaded their motor skills videos into their personal Box (Redwood City, CA) or Dropbox (San Francisco, CA) folder.

### Perceived Competence

We administered the perceived competence assessment, The Self-Perceptions Profile for Adults (α = 0.81–0.92) and Children (α = 0.76–0.91) (46, 47), through Qualtrics (Provo, UT). The Self-Perceptions Profile for Adults and Children (46, 47) domains of athletic competence, physical appearance, and global self-worth were used to assess perceived competence. Qualtrics is an experience management company that specializes in research software and survey development (Provo, UT).

### Beliefs

Semi-structured interviews were conducted, audio-recorded, and transcribed through Zoom (San Jose, CA) to examine beliefs. The Zoom (San Jose, CA) interviews were password-protected to ensure confidentiality.

## Results

The results of this study demonstrate *solution testing* (i.e., feasibility) of the adapted methodology (38).

### Recruitment

A total of 200 families expressed interest in this study, defined as sending an email to the research team or clicking on “Interested in Participation” on the University Research Registry. A total of 76 parent-child dyads consented and assented to be part of this study. Fifteen parent-child dyads dropped out of this study for various reasons: no response, lack of time, and health issues. Sixty-one parent-child dyads completed at least one part of the study.

### Data Collection

For physical activity, a total of 50 parents and 48 children had valid physical activity data, defined as four valid days of wear time (≥10 h of wear time per day) (48, 49). A total of 43 parents had a computed motor competence score, 46 parents completed process measures, 49 parents completed catch percentage, and 44 parents completed maximum jump distance. A total of 45 children had a computed motor competence score, 48 children completed process measures, 49 children completed catch percentage, and 47 children completed maximum jump distance. A total of 57 parents and 49 children completed the perceived competence assessment. A total of 12 purposefully selected parent-child dyads participated in the semi-structured interviews.

### Data Quality

For physical activity, nine accelerometers were lost through USPS mail service and a participant lost one accelerometer. A total of 8 parent-child dyads did not follow the motor competence assessment directions; they did not upload all the motor skills, had problems either filming and/or uploading videos, or their videos that could not be coded. There were 4 parents and 10 children who did not complete the perceived competence assessment accurately. Interviews were not conducted in the parent-child dyad homes, making it challenging to build rapport.

## Discussion

This study demonstrated that conducting online human subject research assessing associations of and beliefs about physical activity, motor competence, and perceived competence is feasible, however, it is important to discuss the challenges we faced.

### Recruitment

The aim of the recruitment methods employed in this study was to reach all eligible participants. Using multiple online platforms for recruitment was done intentionally to have a broad reach across the entire state of Michigan, however the recruitment methods lead to bias. The parent-child dyads' geographic location was centralized to Southeastern Michigan. A few parent-child dyads from Southwestern Michigan, but none from Northern Michigan participated in this study. Also, the sample was majority White, educated, and middle to high socio-economic status (see Table 2). Parents were 88.5% White and children were 75.4% White, parents had high levels of education (52.5% held a graduate degree or higher), and total household income was high (46% was $100,000 and above). This was an exploratory study of online research amid a global pandemic. Thus, conducting this research with this population demonstrates online research is feasible. In terms of application, future recruitment for online research must focus on the inclusion of how to have a wider reach and more diverse populations. For better recruitment methods, recommendations should be taken from online survey researchers (50, 51). Best practice for survey research addresses how to develop sampling methodology, obtain higher response rates, representativeness, and use of quality methodologies (50, 51). Future research must address targeting a more diverse, inclusive, and representative sample to ensure quality and rigor (50).

**Table 2:** Socio-demographic characteristics of parents.

	**Overall % (*n* = 61)**
**Relationship to the child**	
Mother	83.6
Father	16.4
**Age**	
20–29	1.6
30–39	47.5
40–49	47.5
50–59	3.3
**Ethnicity**	
White	88.5
Hispanic or Latino	1.6
Black or African American	3.3
Asian	1.6
Mixed ethnicity	4.9
**Highest level of education**	
High school degree or equivalent	1.6
Some college but no degree	11.5
Associate degree	4.9
Bachelor degree	29.5
Graduate degree or higher	52.5
**Total number of adults in household**	
1	6.6
2	88.5
3	4.9
4	0
≥ 5	0
**Total household income**	
≤ $24,999	9.8
$25,000–$49,999	9.8
$50,000–$99,999	34.4
$100,000–$149,999	23
≥ $150,000	23
**Total number of children in household**	
1	9.8
2	47.5
3	26.2
4	14.8
≥ 5	1.6
**Weight classification**	
Underweight	0
Normal	45.9
Overweight	24.6
Obese	29.5

Another challenge with recruitment was getting participants to consent and assent, and the high rate of dropout (approximately 20%). Expressing interest was defined as sending an email to the research team or clicking on “Interested in Participation” on the University Research Registry. Even after reaching out to interested participants multiple times and through various methods, we could not get a higher rate of participants' consent and assent for participation in this study. Low response rates are common for online research (52), but a low percentage consented and assented even after expressing interest. Lack of study participation or consenting can partially be attributed to technological issues, discussed more below.

### Data Collection

This study utilized numerous online platforms that were challenging for participants to use. Parents had difficulties using the platforms Box (Redwood City, CA) and Dropbox (San Francisco, CA). These platforms required parents first to create an account and then download, upload, and share files. The applications were difficult to use on participants' mobile phones or tablets compared to a computer, but they were continuously being updated and became more user-friendly as the study progressed. In terms of application, utilizing multiple, user-friendly platforms is encouraged for online research (52). Technological challenges have been noted as a problem with conducting research with marginalized populations (53), and technological issues likely contributed to the sample that participated in this study. The two online platforms were complex for parents to use and a potential reason why participants did not consent and assent to the study, dropped out, or did not complete the motor competence assessment. For the most part, parents and children did not have trouble using Qualtrics (Provo, UT) to complete the perceived competence measure. However, the assessment was not fully compatible with this platform. Parent-child dyads did not have any trouble with the application Zoom (San Jose, CA) for the semi-structured interviews.

### Data Quality

A significant challenge of conducting this online study was the lack of online motor competence and perceived competence assessments. The assessment was feasible for parents and children to complete in their homes without the presence of a researcher, however, the created motor competence assessment had significant limitations. The motor competence variable only included four process and two product skills. Therefore, only six measurements were combined to develop an overall motor competence score. The four motor skills did not fully assess the domains of FMS, as there was only one locomotor skill and three ball skills.

Another limitation was test administration. There is no way to assess if participants followed the directions as instructed. This online motor competence assessment was self-administered by parents and children; however, motor competence assessments are generally administered by trained researchers (54). There was no way to assess if parents and children accurately followed the sequence of administration (i.e., watch the multimedia demonstration, complete one practice trial, re-watch the multimedia demonstration, and then complete test trials). This sequence was developed based on the standard protocol for administering the TGMD-3 (39) and has been used in administering product and process skills (41). If parents and children did not follow this sequence, it might have further threatened validity. Also, the quality of the motor skill videos varied. For example, many videos followed the ball rather than the participant's body, making coding challenges. For future research application, an online motor competence assessment needs to be developed that accurately assesses motor competence and considers limitations of participant test administration.

We converted validated paper-based perceived competence assessments to the online platform Qualtrics (Provo, UT) for perceived competence. However, the platform was not ideal. The Self-Perception Profile for Adults and Children consists of a four-choice structured-alternative format (46, 47), and the configuration was not fully compatible. Additionally, there is no way to determine if parents and children who completed the assessment understood the format. These errors may have impacted the perceived competence variable. In terms of application, online platforms, such as Quatrics (Provo, UT), are continually updated and are becoming more compatible with varying assessments. Converted paper-based and in-person assessments should be tested for online validity and reliability.

## Conclusion

The purpose of this study was to describe our process of adapting our study design during the COVID-19 pandemic to account for social distancing and shutdowns, and to examine feasibility and challenges of doing so. This study demonstrated that online research is feasible for examining associations of and beliefs about physical activity, motor competence, and perceived competence and can contribute significantly to advances in scientific knowledge. Important implications of conducting online research include reaching a wider range of participants and being more cost-efficient. Future research must address the challenges this study experienced, specifically focusing on recruitment, technological issues, and assessment methodology. Future research can continue exploring and encouraging motor development in children and parents by addressing these challenges, as this research is critical for supporting low physical activity levels. The ease and accessibility of online research will create endless possibilities for research in motor development.

Due to the COVID-19 pandemic, it appears our society has become accustomed to an online world. Almost everything was conducted online or remotely from work, school, research, doctor visits, food delivery, and even social events throughout the pandemic. As the United States and the whole world slowly emerged from the COVID-19 pandemic, our society will forever be altered, and many things will continue to be conducted online due to ease and accessibility. When feasible, we believe research will continue to have some online or remote component, and this study demonstrated that it is possible. There are many advantages to conducting online research, including wide-reach recruitment methods and ease of participant burden, however, there were numerous challenges that we faced. This research adds to scientific knowledge because it shows the feasibility of adapting data collection to a societal and world-wide event and highlights challenges that must be addressed for future application.

## Data Availability Statement

The data presented in this study are available on request from the corresponding author and must follow the proper data sharing procedures (i.e.., data sharing agreement and human subjects approval).

## Ethics Statement

This study involved human participants and was reviewed and approved by the University of Michigan Ann Arbor, Institutional Review Board. For parent participants, written informed consent was provided. For child participants, written informed consent was provided by the child's legal guardian/next of kin and written assent was provided.

## Author Contributions

This work was part of KS-A dissertation research project. KS-A, RH, AM, TT, and LR: conceptualization, methodology, and writing—review and editing. KS-A: formal analysis, data curation, and funding acquisition. KS-A and LR: writing—original draft preparation. All authors have read and agreed to the published version of the manuscript.

## Funding

This study was funded by University of Michigan Rackham Research Grant.

## Conflict of Interest

The authors declare that the research was conducted in the absence of any commercial or financial relationships that could be construed as a potential conflict of interest.

## Publisher's Note

All claims expressed in this article are solely those of the authors and do not necessarily represent those of their affiliated organizations, or those of the publisher, the editors and the reviewers. Any product that may be evaluated in this article, or claim that may be made by its manufacturer, is not guaranteed or endorsed by the publisher.
